# Totally Implanted Port May Be an Alternative to Centrally Inserted Central Catheter for Measurement of Central Venous Pressure

**DOI:** 10.1155/2020/9180856

**Published:** 2020-06-30

**Authors:** Wei-Ke Kuo, Chih-Yu Huang, Chung-Chieh Yu, Chung-Ching Hua

**Affiliations:** ^1^Division of Pulmonary, Critical Care and Sleep Medicine, Chang Gung Memorial Hospital, Keelung, Taiwan; ^2^Chang Gung University, College of Medicine, Taoyuan, Taiwan

## Abstract

**Background:**

A conventional centrally inserted central catheter (CICC) is frequently used to measure central venous pressure (CVP) to monitor the cardiocirculatory status of patients. The tip of the totally implanted port (TIP) is inserted at the same location in the superior vena cava as that of a CICC, and the TIP has been implanted in many patients with cancer. Measurements of CVP using CICC (CICCP) and TIP (TIPP) may be closely related. *Material and Methods*. Ten patients with TIPs in an intensive care unit were prospectively studied, and 121 records of 4536 paired CICCP and TIPP measurements were collected. A bench test in a static or dynamic setting was performed, and 598 paired measurements taken using CICC and TIP were recorded.

**Results:**

The measurement of TIPP was highly correlated with that of CICCP in patients with cancer, especially those in a calm state. Patients with a calm state and ≥3 consecutive identical TIPP were recorded (≥30 seconds), and 90% of the mean difference between CICCP and TIPP was ≤2 mmHg. The pressure measurements recorded using CICC and TIP were identical in both the static and dynamic bench tests.

**Conclusions:**

TIP may be an alternative to CICC for measuring CVP.

## 1. Introduction

Central venous pressure (CVP) measurements are frequently used to guide fluid resuscitation in critically ill patients [1]. Despite its many limitations, CVP measurement still provides critical information on the cardiocirculatory status of patients [2]. CVP can be measured using a centrally inserted central catheter (CICC), and the ideal location of CICC's tip is the junction of the superior vena cava (SVC) and the right atrium; this location best reduces the risk of thrombosis in patients with cancer [3]. The use of a CICC has been associated with infectious, thrombotic, and mechanical complication rates higher than 15%, and CICC insertion to internal jugular vein and subclavian vein with landmark method can result in arterial puncture, pneumothorax, hemothorax, and hematoma [4]. With ultrasound guidance for internal jugular vein and subclavian vein cannulation, there are increased successful rate and decreased complication such as pneumothorax and artery puncture [5–7]. Ultrasound guidance has been recommended as the standard for internal jugular vein cannulation.

Totally implanted port (TIP) was introduced in the early 1980s, and its safety record and ease of use have made it an integral part of daily clinical practice in oncology [8]. The TIP is designed to enable repeated access to the venous system for the parenteral delivery of medications (especially chemotherapy), fluids, and nutritional solutions and for drawing venous blood samples [9]. The TIP comprises a subcutaneously implanted port (or reservoir) connected to a venous catheter that is most frequently inserted into the internal jugular or subclavian vein, and both its tip and that of the CICC are inserted at the same location, namely, the junction of the SVC and right atrium [3, 9, 10].

CVP measurements made using a peripherally inserted central catheter (PICC) are correlated well with measurements made using a CICC but tend to be higher; the long length and narrow lumen of a PICC may account for this discrepancy [11]. The pressure measurements obtained using the CICC (CICCP) and TIP (TIPP) are similar to the right atrial pressure (RAP) measurements obtained using a Swan–Ganz catheter [12], which is infrequently used in patients with cancer. A TIP has a shorter catheter than a PICC, and its tip targets the same location as a CICC. CVP measurements obtained using the TIP may be closer in value to those obtained using the CICC than those obtained using the PICC or RAP obtained using a Swan–Ganz catheter. Direct comparison of CVP measurements obtained using the CICC and TIP is of considerable interest.

Although there are decreased complications with ultrasound guidance for central vein cannulation, issues remain. First, ultrasound may not be routinely equipped in the intensive care unit (ICU), especially in local hospitals. Second, using ultrasound guidance needs appropriate training for clinicians [6]. If CVP measurements obtained from the CICC and the TIP are closely related, the previously implanted TIP may serve as an alternative to the CICC for measurement of CVP when ultrasound is not available to avoid risk of CICC insertion. In this study, we performed a bench test followed by a prospective clinical study in an ICU to investigate the correlation between CICCP and TIPP.

## 2. Materials and Methods

### 2.1. Catheters and Data Acquisition

A 20-cm Arrow 7-Fr polyurethane two-lumen CICC (Arrow International, Asheboro, NC, USA), two 23-cm single-lumen TIPs (an 8 Fr polyurethane PowerPort [Bard Access Systems, Salt Lake City, UT, USA], and a 7-Fr silicone Arrow Polysite mini-3000 series [Route du Manoir, Ivry Ie Temple, France]) were used in this study.

The catheter of the CICC or TIP was connected to an invasive pressure module (M1006B [Philips Medical Systems, Bӧblingen, Germany]) plugged into a bedside monitoring system (Philips MP60) via a three-way stopcock, high-pressure tubing, and a TruWave disposable pressure transducer (Edwards Lifesciences LCC, Irvine, CA, USA). An MIB/RS232 serial interface card (Philips M8005A_J13) connected the MP60 monitor to a laptop, and the data were transmitted every 10 seconds.

### 2.2. Bench Test

Before commencing the clinical study, the equivalence, pressure gradient, and pressure latency of pressure measurements obtained using the CICC and TIP were tested in vitro.

**Figure 1:**
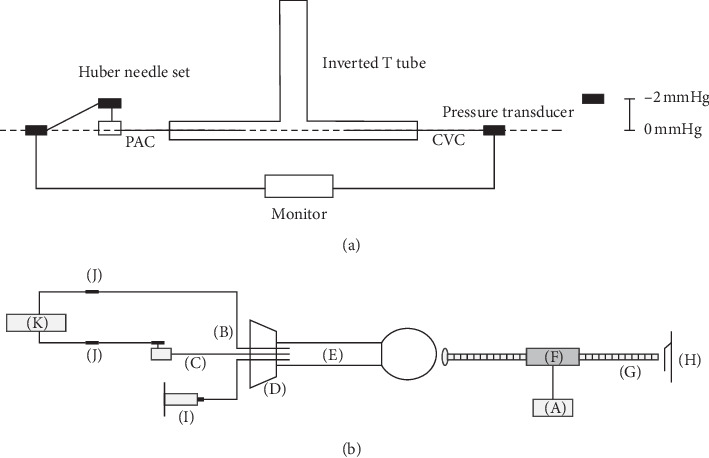
Bench test. (a) Static pressure was measured using an inverted T tube with a CICC connected to one side of the base and one of two TIPs connected to the other through a Huber needle set. Positive pressure and negative pressure were simulated by raising the water level in the vertical part of the inverted T tube and by placing the transducer above the central line of the horizontal part of the inverted T tube, respectively. (b) Dynamic pressure was generated using a piston device developed with an Arduino microcontroller (A). CICC (B) and one of two TIPs (C) were inserted through a stopper (D) into a bulb syringe (E) filled with water. A linear stepper (F) motor with a through-type lead screw (G) controlled by the Arduino microcontroller moved to push the bulb of the syringe to generate pressure at one end and was stopped by a microswitch (H) at the other end. The generated pressure waveforms had amplitudes proportional to the stroke length of the lead screw and frequencies changed by the interstroke pause. Abrupt pressure changes were simulated by adding or withdrawing water using a 10-mL syringe (I) attached to a three-way stopcock that had a tube independently connected to the bulb syringe through the stopper. The pressure in the bulb syringe was transmitted to the HP monitor (K) through the pressure transducers (J) that were connected to the CICC and one of the TIPs.

An inverted T tube with CICC connected to one side of the base and one of 2 TIPs to the other through a Huber needle set (20G x 0.75 in, Bard Access Systems, Salt Lake City, UT, USA) was constructed to test the equivalence of static pressure readings. The static pressure generated was determined by measuring the height of the water column in the vertical tube; the measurement range was −2 to 25 mmHg using the CICC and TIPs (Figure 1(a)).

A pressure-forming piston device developed with an Arduino microcontroller was used to generate waveforms of various amplitudes and frequencies; their baselines were adjusted by adding water to the system or by withdrawing water from the system using a 10 mL syringe (Figure 1(b)). The measured pressure and the range of waveforms generated using the pressure-forming piston device to produce various amplitudes, frequencies, and baselines were recorded, and comparisons were made between the measurement results obtained using the CICC and each of the two TIPs.

Abrupt pressure change was simulated by quickly pushing a certain volume of water into the pressure-forming piston device system. The effect of using an angle from 0° to 180° between the bevel of the Huber needle and the port of the TIP on pressure measurements was also tested.

### 2.3. Clinical Study

#### 2.3.1. Patient Selection

This single-center prospective case series study was conducted at the 10-bed ICU of Chang Gung Memorial Hospital in Keelung, Taiwan, between December 2016 and March 2018. Adult patients with cancer in the ICU who had an implanted TIP with a CICC inserted through the internal jugular or subclavian vein because of clinical requirements were recruited. The brands and specifications of the TIPs and CICC were the same as those used in the bench test. Patients with the following conditions were excluded: continuous infusion of drug or fluid via the TIP or CICC that could not be held for more than 15 minutes, prone position, malposition of the TIP or CICC, resistance to flushing of the TIP or CICC, evidence of catheter-related thrombosis, improper location of the tip of CICC (ideal tip location was in the SVC above the level of the carina on chest X-ray [13]); obvious twisting of the TIP and CICC as evident in a chest X-ray, SVC syndrome, central vein stenosis, or obvious bleeding tendency. This study was approved by the Institutional Review Board of Chang Gung Memorial Hospital (IRB/CGMH, No. 104–9908B). Signed informed consent was obtained from each patient.

#### 2.3.2. CVP Measurement and Data Collection

Patients were positioned in a flat supine position with their arms at the sides of their bodies. The catheters of the CICC and TIP were connected separately to the Philips MP 60 monitor, with the TruWave pressure transducer levelled with the phlebostatic axis [14]. Before each measurement, the catheter was briefly flushed with saline, and data transmission began after the pressure waveforms of CICCP and TIPP stabilized. The pressure readings for both CICC and TIP, heart rate, and respiratory rate were transmitted simultaneously from the monitor to the laptop every 10 seconds and recorded in a 6-minute period for 3–5 times per day until a patient had no further clinical need for monitoring CVP.

The following patient characteristics were recorded: age, sex, brand of CICC/TIP, site of insertion of the CICC/TIP, the internal diameter of the TIP, and the duration of TIP use (days between insertion and the first day on which it was used for pressure monitoring). Whether the patient was in a calm state during the whole six minutes period of measurement (Sedation-Agitation Scale ≦4) [15], whether the patient was receiving sedation (benzodiazepine and opioids), and whether the patient was receiving control or assist control ventilation were recorded in each patient data record.

#### 2.3.3. Parameters

The mean difference of pressure (MDP) between CICCP and TIPP and the coefficient of variation (CV) for the heart rate and respiratory rate (CVHR and CVRR) for each record were calculated. In the bench test, the MDP was assigned to be either static or dynamic depending on whether the pressure was generated by the water in the vertical part of the inverted T tube or by the pressure-forming piston device. For every record in which the patient was in a calm state, a series of differences between simultaneously measured CICCP and TIPP were calculated, and the longest period (LP) in which each of the CICCP and TIPP measurements had a series of identical values individually was identified.

### 2.4. Statistical Analysis

Data were analyzed using SPSS 19.0 (SPSS Software, Chicago, IL, USA). The difference between the MDP of groups was tested using an independent sample t-test. Simple linear regression was used to assess the associations between MDP and patient characteristics, CVHR and CVRR. The correlation between the CICCP and TIPP in each record was analyzed using Spearman's rank correlation coefficients. A *p* value < 0.05 was considered statistically significant.

## 3. Results

### 3.1. Bench Test

**Figure 2:**
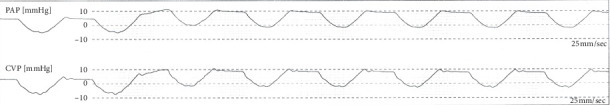
Dynamic pressure waveforms recorded by the CICC (CICCP) and TIP (TIPP) were nearly identical in the simulations of abrupt pressure changes.

Five hundred ninety-eight paired pressure measurements were recorded. Static MDP values between the CICC and each of the two TIPs, as determined by the difference in the height of the water column in the inverted T tube, were all 0 mmHg. Dynamic MDP values between the CICC and each of the two TIPs, as determined using various combinations of pressure (1–43 mmHg; CICC or TIP) and frequency (29–167 cycles/min), were all 0 mmHg. Dynamic MDP values between CICC and each of the two TIPs were all 0 mmHg, and the waveforms recorded using the CICC and TIP were nearly identical (Figure 2) in 10 simulations of abrupt pressure change. The angle between the bevel of the Huber point needle and the port of the TIP did not affect the pressure readings.

### 3.2. Clinical Study

**Figure 3:**
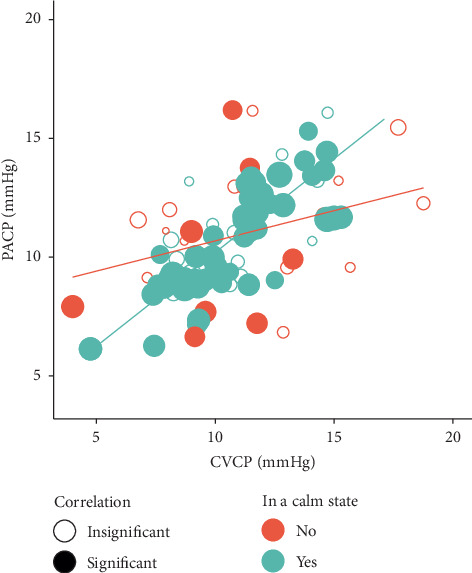
The means and correlations of CICCP and TIPP values in each recording were stratified by the calm state of patients. CICCP and TIPP had the slope of regression line between their means closer to the diagonal line and had more significant correlations in the periods in which patients were in a calm state than those periods in which they were not. A closed circle denotes a significant correlation, and the size of the circle is proportional to the correlation coefficient.

Ten patients were included in an in vivo study, and their clinical characteristics are presented in Table 1. Most patients were male and intubated with endotracheal tube, and all patients were with solid cancer. CICCs were inserted with jugular vein approach by landmark method, and TIPs were inserted with subclavian vein approach by C-arm. One hundred twenty-one records of 4356 paired TIPP and CICCP data points were collected, and their MDP was 1.66 with a standard deviation of 1.54.  Table 2 lists the differences of MDP according to the characteristics of the patients. Patients in a calm state, under sedation, or not receiving control/assist control ventilation had lower MDP values than those who were agitated, not sedated, or receiving controlled ventilation. Patients with the TIP implanted on the right side or a different side from the CICC had higher MDP values than those not exhibiting these characteristics. There were no statistically significant differences of MDP values between different gender and TIP brands.  Table 3 presents the simple linear regression results for MDP. Being in a calm state and respiratory rate variability, as represented by CVRR, were significant predictors of MDP. The scatterplot in Figure 3 indicates that CICCP and TIPP were more significantly correlated, had higher correlation coefficient, and had closer values in the records in which the patient was in a calm state than in those in which the patient was not.  Table 4 lists the differences and correlations between CICCP and TIPP as determined using a cutoff count of identical values in the LP (i.e., 3–6) in the records of patients in a calm state. The paired CICCP and TIPP series with a count of identical values ≥ 3 in the LP had both the highest percentage of a pressure difference ≤2 mmHg and the highest correlation coefficient.

## 4. Discussion

In both static and dynamic bench tests, the pressure measurements recorded using the TIP were equivalent to those recorded using the CICC. TIPP was highly correlated with CICCP in ICU patients, especially those in a calm state. In calm ICU patients, the TIPP in a 30-second period at a stable level may be a surrogate for CICCP. Therefore, the TIP may be an alternative to the CICC for measuring CVP.

Pulse pressure variation (PPV), stroke volume variation (SVV), and passive leg raise test (PLRT) had excellent predictive value for fluid responsiveness. However, PPV and SVV could only be used in patients who were ventilated, sedated, and without arrythmia, and PLRT was not so easy to be performed [2]. Using CVP to guide fluid resuscitation still provides some important information when there is no other choice.

The TIP and PICC are central venous catheters and are essential tools in the management of patients with cancer [16]. TIP, but not PICC, is applied for long-term use and has a low risk of infection [16]. The desired location for placement of the catheter tips of the implanted TIP and PICC is the same as that of a CICC [3, 9, 10, 16], implying they are reasonable alternatives for measuring CVP.

A PICC is pliable, long (approximately 50–60 cm), and narrow (3–5 Fr). CVP measurements obtained using PICCs approximate those obtained using CICCs with the help of a continuous infusion device [11]. PICCs and CICCs do not differ in their ability to transmit static or dynamic pressure in vitro and yielded CVP readings with insignificant differences in a clinical study of 10 patients in which one of them had respiratory failure [17, 18]. A TIP is shorter and has an internal lumen larger than that of a PICC [17, 18]. and its length and size are comparable to those of a CICC. According to Poiseuille's law, the pressure transmission loss by the catheter itself in the TIP tends to be less than that of a PICC and comparable to that of a CICC. This point of view was proved by our dynamic bench study, in which dynamic MDP values were all 0 mmHg between CICC and each of the two TIPs.

A Huber needle set, which has a smaller diameter than that of TIP, is necessary for establishing the connection between a TIP and the pressure transducer. In this study, the small diameter of the Huber needle did not result in discrepant pressure transmission between the TIP and CICC in the bench test. On the other hand, the catheter of the TIP is connected to a small reservoir sealed by a soft silicone on top of Huber needle punctures. The reservoir of the TIP may achieve a pressure balance when the simulator of SVC filled with water following the communicating vessels principle in a bench test; however, this was not the case with SVC in a clinical study where the pressure was everchanging and full of blood, which is non-Newtonian [19]. The difference between TIPP and CICCP measurements in the clinical study may reflect the fluctuating CVP level caused by the complex interaction between cardiac function and venous return [20].

The fluctuation of CVP can be enhanced through transmural pressure change in forced expiration, cardiac rhythm disorders, and respiratory variation [20]. CVP measurement in a clinical setting is not an easy task and may be affected by the zeroing and levelling of the transducer and any minor movement of patients and their respiration [21, 22]. In the present study, TIPP measurements were correlated with those of CICCP, with only small discrepancies in intubated patients with ventilation support, especially those in a calm state. TIPP may be a surrogate for CICCP in calm ICU patients with an already inserted TIP.

Taking single paired measurements in which the hemodynamic status was stabilized for at least 1 hour, Blot et al. determined that RAP, as measured using a Swan–Ganz catheter, was very close to CVP, as measured using a CICC or TIP; this finding suggests that CVP can be accurately measured using a TIP [12]. Although CVP is considered to be a surrogate for RAP [23], using the same location for CICC and TIP tips in SVC can ensure that the same target of pressure measurement is met. Hemodynamic instability is frequently encountered in ICU patients, and the serial paired measurement of CICCP and TIPP may more reflect the reality of clinical practice than single measurement. CVP trends, rather than a single measurement, are critical for hemodynamic management [23, 24]. In the present study, TIPP measurements were correlated with CICCP measurements in calm ICU patients. Calm ICU patients who had 3 consecutive identical TIPP values in 30 seconds had a 90% chance of having a mean difference of ≤2 mmHg between their CICCP and TIPP.

Our study has several limitations. First, in the clinical study, discrepancies were noted between CICCP and TIPP values that were not observed in the bench test. The simulator SVC in the bench test was different from the SVC in diameter, wall elasticity, and the contained fluid. Conducting SVC echography and a blood test at the time of taking each measurement may be helpful for investigating the differences between CICCP and TIPP in the clinical study. Second, the data of CICCP and TIPP measurements were transmitted at a fixed interval of 10 seconds, and they may not have been measured at the base of c or a wave [20]. However, the levels of CICCP and TIPP were captured simultaneously and digitally, and their correlations can reflect their dynamic relationship over 6 minutes. Low sampling frequency may have produced aliasing with respect to the records of heart and respiratory rates, making their CV imprecise. Further study with a high frequency of data collection is necessary for clarifying the effect of heart rate or respiratory stability on CICCP and TIPP measurements.

## 5. Conclusions

For patients with cancer and an already implanted TIP, the insertion of a CICC for measuring CVP may cause additional risks when ultrasound is not available. In bench tests, CICCP and TIPP values were identical. In calm ICU patients, TIPP values were highly correlated with CICCP values in serial measurements and may function as a surrogate for CICCP in a 30-second period at a stable level. Given these points, TIP may be an alternative to CICC for measuring CVP. However, this study was performed at a single center with a small sample size, and a large-scale study is necessary to verify our preliminary results.

## Figures and Tables

**Table 1:** Characteristic of 10 patients with implanted TIP and CICC insertion.

Age, years		59.2 ± 13.4
Gender		
	Male	8
	Female	2
Type of cancer		
	Solid	10
	Hematologic	0
Intubated with endotracheal tube		
	Yes	9
	No	1
TIP brand		
	Powerport	6
	Polysite	4
Side of TIP implantation		
	Left	4
	Right	6
Duration of TIP implantation, days		167.7 ± 133.0
Side of internal jugular vein for CICC insertion		
	Left	4
	Right	6

TIP, totally implanted port; CICC, centrally inserted central catheter.

**Table 2:** The difference of MDP by the characteristics of patients.

		Records	MDP	*p* value
Gender				0.13
	Male	99	1.56 ± 1.58	
	Female	22	2.10 ± 1.28	
TIP brand				0.86
	Powerport	70	1.68 ± 1.76	
	Polysite	51	1.63 ± 1.17	
Side of TIP implantation				<0.01
	Left	34	2.54 ± 1.61	
	Right	87	1.31 ± 1.37	
Side of TIP versus CICC				0.04
	Same	45	1.28 ± 1.32	
	Different	76	1.88 ± 1.62	
In a calm state				<0.01
	Yes	93	1.08 ± 0.91	
	No	28	3.58 ± 1.63	
Under sedation				<0.01
	Yes	100	1.43 ± 1.37	
	No	21	2.74 ± 1.84	
Control/Assist-control ventilator mode				0.02
	Yes	52	1.84 ± 1.70	
	No	59	1.22 ± 1.07	

Data are presented in mean ± standard deviation. CICC, centrally inserted central catheter; TIP, totally implanted port; MDP, the mean difference between pressure measured by CICC and TIP in each record.

**Table 3:** Simple linear regression for MDP.

	*β*	95% CI of *β*	*p* value
Age	0.00	–0.03∼0.02	0.78
Gender	0.09	–0.77∼0.96	0.83
Side of TIP implantation	–0.62	–1.66∼0.42	0.24
TIP and CICC in the same side	–0.38	–0.86∼0.11	0.13
Control/Assist–control mode	–0.29	–0.86∼0.27	0.30
In a calm state	–2.31	–2.87 ∼ –1.76	<0.01
Under sedation	–0.16	–1.45∼1.12	0.80
CVHR	7.15	–1.29∼15.59	0.10
CVRR	–2.73	–5.39∼ –0.08	0.04

*β*, unstandardized regression coefficient; CI, confidence interval; CICC, central inserted central catheter; TIP, totally implanted port; MDP, the mean difference between pressure measured by CICC and TIP in each record; CVHR, the coefficient of variation of heart rate; CVRR, the coefficient of variation of respiratory rate.

**Table 4:** The difference of and the correlations between CICCP and TIPP in the records with subjects in a calm state.

Count of identical values in LP^*∗*^	Record numbers	Percentage of difference between CICCP and TIPP ≤2 mmHg (%)	Spearman's rank correlation	
			Rho	*p* value
≥3	1185	90.0	0.733	<0.001
≥4	888	89.3	0.694	<0.001
≥5	672	86.3	0.701	<0.001
≥6	517	88.6	0.706	<0.001

^*∗*^The data count of the longest episode (LP) in which each of CICCP and TIPP had an individual series of identical values. Series of CICCP and TIPP were included in the analysis if their counts of identical values in LP were equal to or greater than the number listed in the column. CICCP, pressure measured by central inserted central catheter; TIPP, pressure measured by totally implanted port; LP, longest period.

## Data Availability

The data used to support the findings of this study are available from the corresponding author upon request.
